# A Cross-Sectional Study of Risk Factors Affecting Milk Quality in Dairy Cows

**DOI:** 10.3390/ani13223470

**Published:** 2023-11-10

**Authors:** Marios Moschovas, Georgios Pavlatos, Zoitsa Basdagianni, Georgios Manessis, Ioannis Bossis

**Affiliations:** 1 Chrisodima Veterinary Services S.H., Andrea Syngrou Avenue 191, 17121 Athens, Greece; moschovas.xrisodima@gmail.com; 2 Laboratory of Anatomy and Physiology of Farm Animals, Department of Animal Science, School of Animal Biosciences, Agricultural University of Athens (AUA), Iera Odos 75 Str., 11855 Athens, Greece; stud214087@aua.gr (G.P.); gmanesis@aua.gr (G.M.); 3 Laboratory of Animal Husbandry, Department of Agricultural Sciences, School of Agriculture, Forestry and Natural Resources, Aristotle University of Thessaloniki (AUTH), 54124 Thessaloniki, Greece; basdagianni@agro.auth.gr

**Keywords:** mastitis, dairy cows, risk factors, udder lesions, teat lesions, teat hyperkeratosis, milk composition, milk quality, cow welfare

## Abstract

**Simple Summary:**

Modern dairy cattle farms have intensified greatly in recent years to meet the increased global demand for fresh milk. For this reason, health and welfare risks are a great concern to the dairy industry, with mastitis still being among the most significant, as it decreases milk quality and farmer profit. In the present study, 1004 dairy cows from one farm in Greece were included in the study. Data regarding the animals were collected from the farm’s database, while for each cow, health and welfare traits were recorded before collecting milk samples. Each milk sample was tested for somatic cell counts and gross milk composition, and the traits recorded were assessed as potential risk factors affecting milk quality. Higher lactation periods and teat hyperkeratosis seemed to significantly lower milk quality traits and increase somatic cell counts. Udder cleanliness and teat size also had a negative effect on protein content. These results indicate the importance of proper milking routine and hygiene and culling older cows, in improving milk quality. The relevant literature and enhanced milking and management procedures are discussed.

**Abstract:**

Despite years of research devoted to bovine mastitis, the disease remains a serious problem in dairy cattle, causing economic losses to the dairy industry worldwide due to reduced milk yield, lower milk quality, drug costs and early culling of cows. The aim of this study is to determine the importance of several risk factors affecting milk quality in dairy cows, as well as to highlight proper milking techniques. A cross-sectional study was performed in one Greek dairy farm with the inclusion of a total of 1004 Holstein Friesian cows in the study. The udder and teat traits were recorded for each cow, while individual milk samples were used to estimate the somatic cell count (SCC) and gross milk composition. The traits recorded were examined as potential risk factors affecting milk quality using the Akaike information criterion (AIC) and the algorithm stepAIC to select the best linear regression model which explains the data. Overall, the prevalence of mastitis was ca. 9%. With an increase in the lactation period, the SCC increased (*p* ≤ 0.05) while fat (*p* ≤ 0.05), protein (*p* ≤ 0.001) and lactose (*p* ≤ 0.001) content decreased. Teat hyperkeratosis increased the SCC (*p* ≤ 0.05) and decreased P content (*p* ≤ 0.05). Proper husbandry management and milking procedures are considered essential to maintain milk quality of high standards.

## 1. Introduction

In recent years, growing global demand for dairy products has resulted in the intensification of livestock production systems. Although the global dairy cattle population has increased ca. 26.0% in the last 20 years, global milk production has increased more than 51.0% [1]. This implies that farms have had to undergo serious changes in order to meet global demand and achieve the highest milk yield possible, without undermining the cows’ welfare and natural behavior. For cattle, and for the vast majority of farm animals, pasture is a natural environment, allowing them to express normal behavior, lie in stretched positions and reduce the prevalence of diseases compared to indoor housing [2]. Thus, modern intensive dairy farms have had to face the challenge of managing health and welfare issues, like mastitis and lameness, which can challenge their profits in various ways.

Mastitis constitutes a major challenge and the most prominent threat in the dairy sector worldwide, despite the extensive implementation of mastitis control strategies, and remains the most economically important disease of dairy cattle, accounting for 38% of the total direct costs of the common production diseases, while at the same time, it has been estimated as the most common cause of death in adult dairy cows [3]. The annual losses in Europe due to mastitis are over 1 billion euros, whilst mastitis prevalence fluctuates between 8% and 48% per herd [4]. It is exceptionally complicated to estimate the losses affiliated with mastitis, which can be divided among reduced milk production, discarded milk, veterinary services, medication, labor, product quality, materials and investments and culling. The cost of each one of these factors might differ amongst regions, but the economic principles behind them correspond [5]. Bovine mastitis has been defined as an inflammatory response of the mammary gland, usually as a consequence of microbial infection, but can also have a non-infectious etiology [6,7,8] and can be classified into clinical or sub-clinical, chronic or acute and contagious or environmental mastitis, based on the extent and the etiology of inflammation. Clinical bovine mastitis is apparent and easily detected, causing abnormalities to cows’ udders, whilst severe cases might be fatal. Red, swollen udders and fever are some common clinical features of clinical mastitis. Depending on the degree of inflammation, clinical mastitis can be divided into per-acute, acute and sub-acute [8], whereas if the inflammatory process has extended duration with clinical outbursts occurring at irregular intervals, the mastitis case can be described as chronic. On the contrary, sub-clinical mastitis shows no visible abnormalities in the udders or milk; therefore, diagnosis can be extremely difficult. In cases of sub-clinical mastitis, milk production decreases, whereas there is an elevation in the somatic cell count (SCC). Although it is very difficult to estimate the economic losses caused by sub-clinical mastitis, experts agree these are much more than those owing to clinical mastitis. A vast range of organisms, such as bacteria, mycoplasma, yeasts and algae, have been implicated as causes of the disease. Nowadays, the two most common causes of bovine mastitis are *Escherichia coli* and *Streptococcus* spp. and constitute an increasing problem in low somatic cell count herds [7]. One of the many causes of bovine mastitis is deemed to be bacterial intra-mammary infection (IMI). Depending on the pathogens, bovine mastitis has been classified as either contagious or environmental [9]. Contagious pathogens can be transmitted from cow to cow, usually during milking and throughout the milking period, commonly leading to sub-clinical cases. These pathogens can be considered organisms adapted to survive within the host [7] and the most prevalent contagious pathogens are *Staphylococcus aureus* and *Streptococcus agalactiae*, whereas species such as *Mycoplasma bovis* and *Corynobacterium* are less common [8]. *Staphylococcus aureus* is thought to be the most common mastitis pathogen isolated from milk samples. It can be a colonizer on cows without causing inflammation, or it can transmit from cow to cow, turning into sub-clinical mastitis or evolving into clinical mastitis [4]. On the other hand, environmental pathogens do not usually live on the cow’s udder and teat skin and can be described as opportunistic invaders causing clinical mastitis. A wide variety of bacterial species are reported to cause environmental mastitis, such as *Streptococcus* spp., coliforms (*E. coli*, *Klebsiella* spp., *Enterobacter* spp.), *Pseudomonas* spp., *CN Staphylococci*, etc. Pathogens like *Escherichia coli* or *Streptococcus uberis* invade the cow’s udder and multiply, induce a host immune response and are rapidly eliminated.

### 1.1. Risk Factors

Several risk factors have been found able to cause bovine mastitis and must be taken into consideration to prevent such cases. Bacterial intra-mammary infection (IMI) is classified as one of the principal causes of bovine mastitis provoked by a wide variety of bacterial species, which have been mentioned above. Breeding and genetic factors, such as breed, udder anatomy and its structure, should also be taken into consideration, because of their effect on susceptibility and resilience to mastitis. It is reported that high-yielding cows are more vulnerable to mastitis than medium-yielding breeds [10], whereas primiparous cows are more resilient to IMI than multi-parous cows [11]. Moreover, the distance between the teat and the floor, as well as the teat size and teat end lesions, may also increase the prevalence of IMI and the SCC [8,12]. Age also affects susceptibility to mastitis. Due to frequent milking or damage caused by previous inflammations, older cows are less resilient to infections causing both clinical and subclinical mastitis [8,13,14]. Other factors that might affect the resilience to mastitis pathogens are the parturition period and the first month of the lactation period, because of immuno-suppression [15,16,17], as well as insufficient nutrition, which might lead to a negative energy balance and consequently to an increased susceptibility to diseases [18]. Last but not least, proper housing management constitutes one of the most underestimated risk factors for mastitis. Inadequate sanitation, poor ventilation and high humidity are some management oversights that need to be taken into consideration, especially in warm and humid climates, like the Mediterranean [6,14,19].

### 1.2. Prevention and Treatment

The treatment of mastitis remains one of the most challenging issues for the dairy industry. Although the use of antibiotics remains the main approach for therapy, research on treatment alternatives is of utmost importance. One of the most effective ways to control mastitis is proper milking management, which consists of some specific activities, such as treatment of the disease (clinical or subclinical), Dry Cow Therapy (DCT), prevention of transmission of infection (environmentally or from cow to cow) and prevention via the improvement of the immune system [5]. The most common methods to prevent a mastitis outbreak in a herd via efficient management are related to the livestock’s housing conditions. Teat disinfection pre- and post-milking is one of the most important preventive measures in mastitis control, in conjunction with the bedding material [11,20], as well as the ventilation and the temperature of the stall. Despite the cost and the adjacent perils that might occur, antibiotics still constitute a renowned strategy against mastitis. Natural or synthetic, such as penicillin, ampicillin, tetracycline, etc., they can be administered via intramuscular or intravenous injections and intra-mammary infusion [21], usually combined with DCT during the cow’s dry period, in order to prevent severe cases throughout the forthcoming lactation period. Although antibiotics might be effective against mastitis, their overuse can induce numerous threats, not only to the dairy industry and the livestock but also to public health [8]. Finally, vaccination against some of the bacteria mentioned above can be described as a preventive method to treat mastitis. However, this cannot be deemed as the sole way to confront mastitis and should be seen as an adjunct to other control procedures, such as hygienic milking, culling of infected cows and antibiotic treatment [11,14,22].

Despite numerous bodies of research and technological advances that have been made, mastitis continues to be a major issue for the dairy industry [11]. The continuous search for innovative treatment alternatives and control programs is urgent. Most scientific papers concentrate on the effect of parity number and teat end lesions on the SCC and mastitis prevalence, while in this study, other factors were taken into consideration at the same time. In addition, the statistical approach used produced a simple model to evaluate the effect of several factors on milk quality traits and mastitis prevalence, enabling us to better understand the factors that predispose cows to mastitis. The objective of this cross-sectional study was to assess the impact of several risk factors on the SCC and gross milk composition with data retrieved from a large Greek dairy farm, aiming to help with the planning of a more efficient mastitis control program.

## 2. Materials and Methods

### 2.1. Study Population, Assessment of Udder Characteristics and Acquisition of Milking Data

One modern, intensive dairy cow farm, located in Evros in Eastern Macedonia–Thrace, Greece, was involved in the study. The data were collected from 1004 Friesian-Holstein lactating cows, which are housed in different barns on the farm and managed under a similar system. The udder characteristics and health status were assessed by an experienced veterinarian during morning milking throughout the study. More specifically, the udder’s size (US, 4-scale score;1 = small, 2 = medium, 3 = normal, 4 = large), cleanliness (UC, 4-scale score; 0 = clean, 1 = slightly dirty, 2 = dirty, 3 = very dirty), the oedema caused by clinical mastitis (CM, 2-scale score; 0 = healthy, 1 = mastitis), teat size (TS, 3-scale score; 1 = small, 2 = normal, 3 = large) and teat hyperkeratosis (TH, 4-scale score; 0 = no ring, 1 = smooth or slight ring, 2 = rough ring, 3 = very rough ring with cracks) were recorded for each individual cow. Each animal was assigned a single score for TH using the highest recorded score among the animal’s teats (4-scale score; 0 = no hyperkeratosis, 1 = at least one teat with score 1, 2 = at least one teat with score 2, 3 = at least one teat with score 3). Accordingly, the cows were assigned a single value for mastitis (0 = healthy, 1 = cows having symptoms of clinical mastitis in at least one quarter) and teat size (3-scale score; 1 = at least one small teat, 2 = all teats normal, 3 = at least one large teat). Data, including ear tag number, lactation period (LP, 5-scale score; 1st, 2nd, 3rd, 4th and ≥5th lactation), days in milk (DIM), daily milk yield (DMY) and bedding type (2-scale score; 1 = bed housing system, 2 = free stall compost barn), were also collected for each individual cow using the farm’s electronic database. 

### 2.2. Milk Sampling and Analysis

The milk samples were collected during one farm visit in October 2022 from each individual cow and transferred immediately to the farm’s laboratory for chemical analyses. Sample collection was performed following the ICAR (International Committee of Animal Recording) recommendations. More specifically, one milk sample (ca. 70 mL) was collected per cow during the morning milking. The samples were transferred to a laboratory at 4 °C and were analyzed within 24 h. Milk analyses included somatic cell counts (SCCs) (Fossomatic™, Foss, Hilleroed, Denmark) as well as fat (F), protein (P), lactose (L), solid-non-fat (SNF) and total solids (TS) content (MilkoScan™, FT+, Foss, Hilleroed, Denmark).

### 2.3. Statistical Analysis

Statistical analysis was conducted both in the R3.6 (https://www.r-project.org/, accessed on 10 April 2023) and SPSS v23 software (IBM Corp., Armonk, NY, USA). The statistical significance threshold was set at 0.05. Mastitis prevalence was calculated as the percentage of affected animals of the total population under study. Descriptive statistics (mean ± standard deviation) for MY, SCC, F, P, L, SNF and TS were also calculated.

To test the effect of housing, DMY, DIM, lactation period, mastitis prevalence, udder size, udder cleanliness, teat size and teat hyperkeratosis on the SCC, F, P, L, SNF and TS, a multiple regression model was used. The fitted regression model was:

Y_ijklmnop_ = *μ* + H_i_ + LP_j_ + M_k_ + US_l_ + UC_m_ + TS_n_ + TH_o_ + *a*_1_^Χ^ DMY + *b*_2_^Χ^ DIM + e_ijklmnop_

where Y_ijklmnop_ = dependent variables (SCC, F, P, L, SNF, TS), *μ* = the overall mean, H_i_ = the effect of housing (i = 2 levels), LP_j_ = the effect of the lactation period (j = 5 levels), M_k_ = mastitis prevalence (k = 2 levels), US_l_ = udder size (l = 4 levels), UC_m_ = udder cleanliness (m = 4 levels), TS_n_ = teat size (*n* = 3 levels), TH_o_ = teat hyperkeratosis (o = 4 levels), *a*_1_ = the linear regression coefficient of DMY, *b*_2_ = the linear regression coefficient of DIM and e_ijklmnop_ = the error term.

In this work, the simplest linear regression model that could explain the data and the observed variations were selected using the Akaike information criterion (AIC) and the algorithm stepAIC in R. To do so, the dataset was split into two sets: the first set (75% of the data) was used to train the stepAIC algorithm, and the second set (25% of the data) was used to assess the performance of the simplest model, which was generated after the application of the stepAIC algorithm. The simplest model was considered the one with the lowest AIC values.

## 3. Results

### 3.1. Mastitis Prevalence and Descriptive Statistics

A total of 1004 Holstein Friesian cows were examined during the present study. The prevalence of clinical mastitis was 9.5% (95/1004). Almost half the population (49.7%, 499/1004) was housed in beds, whereas the other half (50.3%, 505/1004) was housed in a free stall system. The average MY was 32.5 (95% CI 31.86 to 33.18) and the average SCC was 526.76 (95% CI 470.69 to 582.82). Concerning milk quality traits, the average F, P, L, SNF and TS were 4.5 (95% CI 4.42 to 4.56), 3.3 (95% CI 3.30 to 3.36), 4.7 (95% CI 4.69 to 4.73), 8.9 (95% CI 8.85 to 8.92) and 13.4 (95% CI 13.32 to 13.49), respectively. The majority of the studied population of cows were in their first (36.9%, 370/1004) and second (31.0%, 311/1004) lactation periods. Additionally, 15.8% (159/1004), 8.8% (88/1004) and 7.6% (76/1004) of the studied cows were in the third, fourth and ≥ fifth lactation period, respectively. Concerning the lactation stage, 21.2% (213/1004) of the cows were under 50 DIM, 33.7% (338/1004) were between 51–180 DIM and 45.1% (453/1004) were above 181 DIM. Of the studied cows, 27.2% (273/1004), 43.5% (437/1004), 24.9% (250/1004) and 4.4% (44/1004) had a very small, small, normal and large udder size, respectively. The cows with clean udders constituted 25.1% (252/1004), while 32.7% (328/1004) had slightly dirty udder, 23.7% (238/1004) had dirty udders and 18.5% (186/1004) had very dirty udders. Concerning teat hyperkeratosis, 72.7% (730/1004) of the cows had no teat hyperkeratosis, while 11.8% (118/1004), 13.2% (133/1004) and 2.3% (23/1004) had at least one teat with teat hyperkeratosis 1, 2 and 3, respectively. Finally, the majority of cows had a normal teat size (55.6%, 558/1004), while 28.5% (286/1004) had small teats and 15.9% (160/1004) had large teats.

### 3.2. SCC

The linear regression model for SCC is summarized in Table S1. DMY, lactation period, mastitis prevalence and teat hyperkeratosis were found statistically significant in the SCC model. 

After the application of the stepAIC algorithm, the simplest model predicting the SCC was the following:

*SCC* = *DMY* + *lactation period* + *mastitis prevalence* + *teat hyperkeratosis*

The AIC value for the simplified model was 12,705. The estimates and *p*-values of the simplified model are presented in Table S2. The Pearson residuals of the simplified model were symmetrically distributed around zero (Figure S1), indicating a good fitting of the data to the model. The association between the predicted values of the SCC and the variables of the simplified model are schematically presented in Figures S2 and S3. The statistical significance level for the 95% CIs for all graphs was set at α = 0.05.

### 3.3. Fat

The linear regression model for F is summarized in Table S3. The lactation period and DIM were found statistically significant variables in the linear regression model for F.

After the application of the stepAIC algorithm, the simplest model predicting F was:

*F* = *Housing* + *DIM* + *lactation period* + *udder cleanliness*

The AIC value for the simplified model was 2903.4. The estimates and *p*-values of the simplified model are presented in Table S4. The Pearson residuals of the simplified model were symmetrically distributed around zero (Figure S4), indicating a good fitting of the data to the model. The correlations of the predicted values of F to the variables of the simplified model are schematically presented in Figures S5 and S6. The statistical significance level for the 95% CIs for all graphs was set at α = 0.05.

### 3.4. Protein

The linear regression model for P is summarized in Table S5. Housing, lactation period and DIM were found statistically significant variables in the linear regression model for P.

After the application of the stepAIC algorithm, the simplest model predicting P was:

*P* = *Housing* + *DIM* + *lactation period* + *udder cleanliness* + *teat size* + *teat hyperkeratosis*

The AIC value for the simplified model was 836.06. The estimates and *p*-values of the simplified model are presented in Table S6. The Pearson residuals of the simplified model were symmetrically distributed around zero (Figure S7), indicating a good fitting of the data to the model. The associations between the predicted values of *p* and the variables of the simplified model are schematically in Figures S8–S10. The statistical significance level for the 95% CIs for all graphs was set at α = 0.05.

### 3.5. Lactose

The linear regression model for L is summarized in Table S7. Housing, DMY, DIM and lactation period were found statistically significant in the L model.

After the application of the stepAIC algorithm, the simplest model predicting L was:

*L* = *Housing* + *DMY* + *DIM* + *lactation period*

The AIC value for the simplified model was 235.05. The estimates and *p*-values of the simplified model are presented in Table S8. The Pearson residuals of the simplified model were symmetrically distributed around zero (Figure S11), indicating a good fitting of the data to the model. The associations between the predicted values of L and the variables of the simplified model are schematically presented in Figures S12 and S13. The statistical significance level for the 95% CIs for all graphs was set at α = 0.05.

### 3.6. Solid Non-Fat (SNF)

The linear regression model for SNF is summarized in Table S9. Lactation period, DMY and udder cleanliness were found statistically significant variables in the linear regression model for SNF. 

After the application of the stepAIC algorithm, the simplest model predicting SNF was:

*SNF* = *Housing* + *lactation period* + *DMY* + *udder cleanliness* + *teat size* + *teat hyperkeratosis*

The AIC value for the simplified model was 1337. The estimates and *p*-values of the simplified model are presented in Table S10. The Pearson residuals of the simplified model were symmetrically distributed around zero (Figure S14), indicating a good fitting of the data to the model. The associations between the predicted values of SNF and the variables of the simplified model are schematically presented in Figures S15–S17. The statistical significance level for the 95% CIs for all graphs was set at α = 0.05.

### 3.7. Total Solid

The linear regression model for TS is summarized in Table S11. Lactation period, DIM, udder cleanliness, teat size and teat hyperkeratosis were found statistically significant variables in the linear regression model for TS.

After the application of the stepAIC algorithm, the simplest model predicting TS was:

*TS* = * Lactation period* + *DIM* + *mastitis prevalence* + *udder cleanliness* + *teat size* + *teat hyperkeratosis*

The AIC value for the simplified model was 2889.2. The estimates and *p*-values of the simplified model are presented in Table S12. The Pearson residuals of the simplified model were symmetrically distributed around zero (Figure S18), indicating a good fitting of the data to the model. The associations between the predicted values of total solids and the variables of the simplified model are schematically presented in Figures S19–S21. The statistical significance level for the 95% CIs for all graphs was set at α = 0.05.

## 4. Discussion

According to the results from the present study, the SCC was negatively affected by DMY and positively by the lactation period, mastitis prevalence and teat hyperkeratosis. The fat content was negatively affected by the lactation period and DIM, while the protein content was positively affected by housing and DIM and negatively affected by the lactation period, udder cleanliness, teat size and teat hyperkeratosis. The lactose content was also negatively affected by housing, DIM and the lactation period and positively by DMY. Furthermore, the lactation period, udder cleanliness, teat size and teat hyperkeratosis negatively affected the SNF and TS content.

The Akaike information criterion (AIC) was used as an estimator of the prediction error and consequently was exploited to simplify the linear regression models and assess the quality of those simplified models. Using statistical models to explain the processes that generated the observed dataset inherently leads to the loss of some information, as data representation via models is practically never exact. The AIC is based on information theory and can be used to assess the relative amount of information lost through the model, thus dealing with the trade-off between the simplicity and goodness of fit of statistical models. To put it simply, the AIC’s utility lies in its capability to deal with both the risks of overfitting and underfitting the models. In the present work, the AIC was used to produce simple and robust models to evaluate the effect of several factors (udder and teat morphology and health, farming practices and lactation) on mastitis prevalence and milk quality.

Several factors related to the environment, the microflora and each cow’s health and welfare traits have been identified as risk factors of mastitis. Despite the efforts to control mastitis, it remains one of the most important diseases in the dairy industry. The difficulties encountered in controlling mastitis successfully under field conditions reflect both the complex etiology of the disease and a need to improve the control programs. Mastitis control can be improved using sanitation, antibiotic treatment and proper milking procedures. Pathogens are implicated increasingly as important causes of mastitis in dairy herds. The prevalence of mastitis in dairy cows varies, depending on the geographical region and housing environment. In Europe and North America, the prevalence of clinical mastitis in dairy cows ranges from 7% to 30% [23,24,25]. The prevalence rate for clinical mastitis in Ethiopia [26,27] ranged from 3.9% to 25.1% [28]. Additionally, in a study conducted in South Korea, the occurrence of clinical mastitis was 3.7% [29], lower than in other subtropical regions, which have a clinical mastitis occurrence as high as 18.04% [17]. Globally, *Staphylococci* are the most common mastitis-causing agents in cows, followed by *Streptococci* and *E. coli* [25]. In South Korea, among the various organisms isolated from milk samples, the coagulase-negative *Staphylococci* percentage was 44.4%, followed by *E. coli* (17.5%), *Staphylococcus aureus* and *Streptococcus* spp. (14.3% each). Other isolated organisms were Gram-negative microbes of *Salmonella* spp. (6.4%) and *Pseudomonas* spp. (3.2%). However, in the present research, the etiology of mastitis was not studied.

In our study, a positive association was found between mastitis prevalence and SCC (*p* ≤ 0.001). This result is in alignment with the literature and the consensus that mastitis prevalence is associated with high SCC rate; as in previous studies, cows with an elevated SCC were considered to have mastitis [30,31]. In particular, a quarter was determined to have mastitis if the SCC exceeded 300,000/mL, and a cow was diagnosed with mastitis if at least one quarter had a SCC 300,000/mL or higher [32]. However, this threshold of 300,000 cells/mL, as recommended by Klastrup and Madsen (1974), may be high [32]. The SCC of a cow that is not affected by mastitis is <200,000 cells/mL, while at the quarter level, a threshold of <100,000 cells/mL has been introduced for a healthy teat quarter [33,34]. According to recent literature, the clinical mastitis prevalence in dairy cattle peaks around parturition and early in the dry period (the internal period between two lactations in which the cow is not being milked) [35]. Green et al. (2007) reported that older cows with three or more lactations have a higher prevalence of subclinical and clinical mastitis during middle and late lactation, whereas primiparous cows have a higher prevalence of mastitis right after parturition [36]. In this research, the mastitis prevalence was negatively associated with the lactation stage (as lactation progressed, the mastitis prevalence decreased). This result is in close alignment with other studies that reported a higher infection rate during the early lactation stage, compared to mid and late lactation [26,27,29]. The reason for this may be attributed to the physiology of the animals, because in the early stage and due to more oxidative stress and low antioxidant defense, cows are more susceptible to infections [17]. On the other hand, in studies of beef cows, Newman et al. (1991) and Duenas et al. (2001) reported a higher prevalence of IMI at the end of lactation [37,38]. Concerning the lactation period and its association with mastitis in this study, the prevalence of mastitis increased in cows with higher lactation periods. The rate of cows affected by mastitis during the first lactation was considerably lower (2.7%) than the cows during their fifth or more lactation (27.6%). A higher prevalence of mastitis with advancing age has been reported in other studies [26,29,39,40]. An explanation for this, given by Radostitis et al. (2007), is that older cows have larger teats and more relaxed sphincters; thus, the accessibility for infectious agents in the udder is increased [41]. Although Waller et al. (2014), Duenas et al. (2001) and Paape et al. (2000) [38,42,43] did not report a correlation between the prevalence of mastitis and age, they found that mastitis prevalence was augmented as parity increased [26,39,44].

Another important risk factor that was assessed in the present study was the age of the cows (lactation period) and the stage of lactation (DIM). Specifically, a statistically significant negative association was found between the lactation period and fat (*p* ≤ 0.05), protein (*p* ≤ 0.001), lactose (*p* ≤ 0.001), SNF (*p* ≤ 0.001) and TS (*p* ≤ 0.001) content, whereas the same variable was positively associated with the SCC (*p* ≤ 0.05). In addition, two negative associations were found between DIM and fat content (*p* ≤ 0.001) and DIM and TS content (*p* ≤ 0.05). It is reported that age has a significant effect on milk protein and composition in cows [45,46], as well as on fat [46], lactose, SNF and TS comprehensiveness. The milk protein percentage declines in cows older than 3 years old, due to udder tissue deterioration. Kroeker et al. (1985) have also suggested that immunoglobulins increase in conjunction with advancing age, thus affecting the protein content [47]. However, in other studies, an increase in protein and fat yield has been reported in older animals [48,49,50]. Concerning the SCC, in the present study, a positive association was found between the SCC and lactation period. This comes in accordance with numerous bodies of research [48,50,51,52,53,54,55,56] which have reported that multiparous cows have a higher SCC than primiparous cows. On the contrary, Borkowska (2010) found a negative correlation between age and SCC [49]. In early lactation, nutrition may affect milk quality due to a physiological reduction in the capacity of the cow for dry matter intake. Auldist et al. (1995) reported a tendency for an increase in milk fat throughout lactation, explaining that as lactation progresses and the milk yield decreases, the energy balance improves, permitting the synthesis of milk fat [31]. This result comes in contrast with our study, where the fat and TS in milk were higher at the commencement of lactation. The changes in energy balance that occur throughout lactation may have been important in determining the extent of the variation in the fat and TS yield. However, numerous researchers have reported a reduction in milk fat and TS throughout the lactation stages [48,57,58,59,60]. Greater milk production in genetically improved cows has been attributed to increased homeorhetic coordination, the use of animal-tissue-derived nutrients in early lactation and greater proportions of dietary energy partitioned toward milk production [61]. Furthermore, in early lactation, dairy cows have greater proportions of preformed fatty acids, due to increased adipose tissue mobilization [59]. Auldist et al. (1995) also found an increase in the SCC in late lactation, probably due to mammary gland degeneration and the increased permeability of the alveolar epithelium [31]. A similar association was not found in the present study.

Concerning daily milk yield, it was negatively associated with the SCC (*p* ≤ 0.001) and SNF (*p* ≤ 0.001), and positively associated with lactose yield (*p* ≤ 0.001). It is well known that there is a linear correlation between an increasing SCC and milk production, whilst milk production loss can be presumed by the SCC rate [62,63]. A correlation between milk yield and milk quality has been also mentioned before [64], while Nickerson (1995) has reported a minor association between the DMY and SCC and lactose yield, mentioning that heavier breeds tend to produce more milk, but with lower percentages of milk constituents [65]. In addition, Cinar et al. (2015) reported a negative correlation between the SCC and milk yield and lactose, as cows with a low SCC rate are healthier than those with a high SCC rate [66]. Further research on this matter is required.

According to numerous research works, the prevalence of mastitis or IMI indicates that large funnel-shaped teats, pendulous udders and blind quarters lead to udder health issues. In a recent study on beef cows, it was found that cows with blind quarters had severe problems with subclinical mastitis and IMI, while cows that had large funnel-shaped teats or pendulous udders after calving were more susceptible to mastitis and/or IMI. In accordance with another study, the udder depth and teat end shape were associated with the health status of udders. In the same study, which was conducted in the USA, it was found that out of 505 Holsteins and 489 Jerseys, 10% were culled for udder problems. Furthermore, 10% of the cows were sold due to mastitis and an additional 32% were sold due to low milk production, some of which was caused by mastitis [67]. It is believed that selection based on udder and teat morphology may augment efforts to control mastitis. In fact, deeper udders have a higher SCC and are more susceptible to mastitis [68]. In the present study, the udder and teat sizes were mainly normal and no correlation was found between udder size and any of the risk factors, whereas the teat size was negatively associated with protein (*p* ≤ 0.001), SNF (*p* ≤ 0.01) and TS (*p* ≤ 0.05) content. Rathore and Scheldrahe [69] concluded that the teat orifice and the size of the streak canal are natural barriers of bacteria, preventing entrance into the teat cistern. They found that higher stretchability was associated with funnel-shaped teats, larger diameter teats, a faster milk-flow rate and higher-yielding cows [69]. Moreover, it was found that the infected mammary quarters had larger mean diameters of their canals than uninfected quarters, while Seykora (1985) [67] concluded that the location of the teat canal orifice had no relation with the prevalence of subclinical mastitis. In another study, it was found that dairy cows with small teat cistern diameters and shorter canals were predisposed to mastitis [25,70]. Concerning teat hyperkeratosis, the stretching of the teat skin when the liner collapses results in hyperplasia (excessive keratin growth). Teat hyperplasia is a normal physiological response to the forces applied to the teat skin during milking. The onset and severity of hyperkeratosis are profoundly influenced by climate, seasonal and environmental conditions, milking management, herd milk production level and the genetics of individual cows [71]. In general, teat end scores are lower for long pointed teats, slow-milking cows or high-yielding cows [72]. According to Neijenhuis et al. [73], there is a correlation between teat end hyperkeratosis and clinical mastitis caused by *Klebsiella pneumoniae* and *E. aerogens*. Bhutto et al. [74] reported a correlation between teats with hyperkeratosis and the growth of *S. aureus*, coagulase-negative staphylococci, *S. uberis*, *S. agalactiae* and *E. coli*. Some researchers report that teat hyperkeratosis did not have any effect on mastitis prevalence or SCC [75,76], but most of them agree that teat hyperkeratosis leads to an increased SCC [54,55,77,78]. These results come in accordance with our study, where a positive correlation was found between teat hyperkeratosis and the SCC (*p* ≤ 0.001), which is an indicator of mastitis. As aforementioned, 72.7% of the population had no evidence of teat hyperkeratosis, though the etiological agents (pathogens) were not studied. In addition, a genetic correlation has been found between mastitis prevalence and teat hyperkeratosis [79]. Furthermore, in our research, teat hyperkeratosis was adversely associated with SNF yield (*p* ≤ 0.05) and TS yield (*p* ≤ 0.05).

According to Seykora et al. (1985), udder conformation is heritable [80]. Udder depth, udder support, teat placement and udder quality, as well as teat end shape, have been summarized as the genetic factors associated with mastitis in Holstein cows [67]. It was also recorded that cows with lighter skin pigmentation of the udders and teats are more susceptible to teat skin irritation in harsh environments. Furthermore, the correlation of milking speed and infections has been reviewed, but the aspects are divergent. Brown et al. (1986) examined the association between clinical mastitis and milking rate and found a non-significant trend [68], while in two other studies, it was found that high milking rates and a large teat canal diameter had a significant association with an increased SCC [67,81] or the risk of intramammary infections [82]. The heritability of teat-end-to-floor distance, as well as its association with mastitis, makes this trait a selection index for breeding [67,81,83]. Moreover, the interactions between the host’s immune system and pathogens seem to be of particular relevance in the etiopathogenesis of mastitis [84]. It was recently documented that E. coli-infected mammary gland tissues were found to up-regulate the expression of genes related to immune responses, and down-regulate the genes related to fat metabolism [85]. While most risk factors associated with the management and the environment are based on proper hygiene practices, selecting dairy cows which are more resilient to mastitis is also an important management measure.

In order to confront the problem of bovine mastitis in dairy cattle, many researchers support that the best approach is prevention, via proper milking management and hygiene practices. Breeding environment, herd size, feeding, milking technology and hygiene management are some risk factors of utmost importance and must be taken into consideration to eliminate and control mastitis in dairy herds. According to many studies, a significant association has been found between clinical mastitis and farm management, as cows in loose housing systems had a higher risk of clinical mastitis than cows in tie stall housing systems [26,39,86,87]. On the contrary, Gordon et al. (2013) and Valde et al. (1997) reported that tie-stall-housed herds had a significantly higher clinical mastitis rate than free-stall-housed herds [83,88]. As mentioned above, in the present research, 49.7% of the studied population was housed in a bed housing system and 50.3% in a free stall housing system. Poor hygiene also constitutes a risk factor for udder health problems [43]. Several factors can potentially affect cow and udder cleanliness, including housing systems, as cows housed in smaller cubicles tend to be dirtier [89]. It is also known that fecal consistency is correlated with the cleanliness of cows [90]. In our study, a significant rate of cows had very dirty udders (18.5%). The rate of mastitis prevalence in the bed-housed population (4%) was significantly lower than in the free-stall-housed population (14.9%). Specifically, the udder cleanliness (i.e., dirty udders) was negatively associated with the protein (*p* ≤ 0.001), SNF (*p* ≤ 0.05) and TS (*p* ≤ 0.05) content in cow milk. This concurs with the results of Schreiner and Ruegg (2003) and Santman-Berends et al. (2016), where poor udder cleanliness was correlated with increased levels of milk pathogens [91,92] and Reneau et al. (2005), who found that poor udder cleanliness was positively associated with an increased SCC [93]. Although no correlation between the SCC and udder cleanliness was found in the present study, as stated before, the high rate of SCC is an indicator of mastitis, which in turn results in the degradation of milk quality. In a Norwegian study, it was reported that clean udders showed lower values of SCC. These results suggest that udder cleanliness is not merely an aesthetic issue and is closely correlated with udder health and consequently milk quality traits [32]. This study demonstrated that the animal housing system was negatively associated with lactose (*p* ≤ 0.05), meaning that free stall cow milk had a lower lactose content compared to that from cows in bed housing. Regarding milking practices, it is recorded that bucket milking, as well as the absence of teat-cleaning materials before and after milking, seem to be associated with higher clinical mastitis prevalence [83]. Actually, research conducted in Spain has proved the importance of pre- and post-dipping and has correlated the usage of paper towels with low bulk tank SCC, in contrast to cloth towels [94]. The importance of using a towel was also studied by Zeryehun et al. (2013) and Radostits (2007), who recorded that using a towel before and after milking had fewer chances (62.9%) to induce mastitis, compared to not using one (79.7%) [26,41]. Furthermore, Gordon et al. (2013) highlighted the importance of clean bedding material, especially at calving [83]. It is evident that poor bedding hygiene can be a hotbed for environmental pathogens, leading to an IMI outbreak. Another study proposed that inadequately functioning milking machines might cause teat lesions due to pulling or squeezing the teat tissue, with the lining of the streak canal coming out of the teat orifice [67]. Sieber and Farnsworth (1984) also found that milking equipment can cause smooth-ring lesions, which can deteriorate due to milking a dry teat, overmilking or improper handling [95]. Concerning teat health, in the present study, teat hyperkeratosis was studied and only 27.3% of the population had at least one teat affected. Another management factor that should be taken into consideration is dry cow therapy. The importance of the dry period and its correlation with IMI has been studied extensively [96,97,98,99]. According to [26], the absence of dry cow therapy usually leads to high mastitis prevalence at early lactation and early infection, due to the delayed diapedesis of neutrophils into the mammary gland. Cows who were not treated during the dry period were affected at a higher rate (95.9%) than those who received treatment (69.9%). This could be due to the low bactericidal and bacteriostatic quality of milking during the dry period, as well as the incapability of the quarter to generate phagocytic and bactericidal activity [100]. Bradley et al. (2000) reported that mammary glands infected during the dry period were at a higher risk of clinical mastitis, while cows with an IMI in previous dry periods were affected faster by clinical mastitis after calving [101]. Thus, the control of IMI during the dry period can affect the occurrence of clinical mastitis after calving [26,102].

The treatment of mastitis remains one of the most challenging issues for the dairy industry and its control can be achieved via proper management techniques. According to the National Institute for Research [20], a Five Point Plan (FPP) has been introduced since 1960, making important progress in the control of mastitis using a simple and efficient plan which can be easily communicated to every dairy farmer. The appliance of the FPP resulted in a decreased prevalence of clinical mastitis and contagious pathogens [103]. The Five Point Plan consists of five simple steps: (i) identification and treatment of clinical cases, (ii) post-milking teat disinfection, (iii) dry cow therapy, (iv) culling chronic cases, (v) maintenance of the milking device. Bramley (1984) recorded that a control program based on post-milking teat disinfection and drying therapy would diminish the prevalence of infection by 50% within a year [104]. Although the FPP is still successful up to a point, the increased importance of environmental pathogens has led researchers and farmers to study and combine numerous environmental management methods, in order to control mastitis on farms [103]. 

## 5. Conclusions

The application of preventive protocols and proper milking management is an important part of mastitis control and milk quality improvement. The continuously high prevalence of clinical or subclinical mastitis and IMI in numerous herds indicates that milk quality risk factors need to be studied more extensively. Cows with a higher lactation period and cows with severe teat hyperkeratosis were found to have a higher SCC and poorer milk quality traits. This indicates the importance of management practices and udder health in association with mastitis prevalence and milk quality. The treatment methods and prevention techniques of mastitis need to be ameliorated, whilst reducing the use of antibiotics and preserving cows’ health and welfare.

## Data Availability

The data presented in this study are available within the article and Supplementary Materials. Raw data supporting this study are available from the corresponding author upon reasonable request.

## Supplementary Materials

The following supporting information can be downloaded at https://www.mdpi.com/article/10.3390/ani13223470/s1: Table S1: Association between housing, lactation period, days in milk, daily milk yield, mastitis prevalence, udder size, udder cleanliness, teat size, teat hyperkeratosis and somatic cell count; Table S2: Association between somatic cell count and daily milk yield, lactation period, mastitis prevalence and teat hyperkeratosis after using stepAIC; Table S3: Association between fat content and housing, lactation period, days in milk, daily milk yield, mastitis prevalence, udder size, udder cleanliness, teat size and teat hyperkeratosis; Table S4: Association between fat content and housing, days in milk (DIM), lactation period and udder cleanliness after using stepAIC; Table S5: Association between protein content and housing, lactation period, days in milk, daily milk yield, mastitis prevalence, udder size, udder cleanliness, teat size and teat hyperkeratosis; Table S6: Association between protein content and housing, days in milk (DIM), lactation period, udder cleanliness, teat size and teat hyperkeratosis after using stepAIC; Table S7: Association between lactose content and housing, lactation period, days in milk, daily milk yield, mastitis prevalence, udder size, udder cleanliness, teat size and teat hyperkeratosis, Table S8: Association between lactose content and housing, daily milk yield (DMY), days-in-milk (DIM) and lactation period after using stepAIC.; Table S9: Association between solid non-fat content and housing, lactation period, days in milk, daily milk yield, mastitis prevalence, udder size, udder cleanliness, teat size and teat hyperkeratosis; Table S10: Association between solid non-fat content and housing, lactation period, daily milk yield (DMY), udder cleanliness, teat size and teat hyperkeratosis after using stepAIC; Table S11: Association between total solid content and housing, lactation period, days in milk, daily milk yield, mastitis prevalence, udder size, udder cleanliness, teat size and teat hyperkeratosis; Table S12: Association between total solids content and lactation period, days in milk (DIM), mastitis prevalence, udder cleanliness, teat size and teat hyperkeratosis after using stepAIC; Figure S1: Pearson residual distribution of the simplified model for SCC; Figure S2: A. Positive association between SCC and mastitis prevalence. The slope A for mastitis prevalence was 953.99. The gray area represents the 95% CI of SCC values vs. the mastitis prevalence values, and B. Positive association between SCC and teat hyperkeratosis. The slope B for teat hyperkeratosis was 126.12. The gray area represents the 95% CI of SCC values vs. teat hyperkeratosis values; Figure S3: A. Positive association between SCC and lactation period. The slope A for lactation period was 157.84. The gray area represents the 95% CI of SCC values vs. the lactation period values. B. Negative association between SCC and DMY. The slope B for DMY was −25.29. The gray area represents the 95% CI of SCC values vs. DMY values; Figure S4: Pearson residual distribution of the simplified model for fat content; Figure S5: A. Positive association between fat content and housing. The slope A for housing was 0.19. The gray area represents the 95% CI of fat content values vs. the housing values. B. Negative association between fat content and DIM. The slope B for DIM was −0.002. The gray area represents the 95% CI of fat content values vs. DIM values; Figure S6: A. Negative association between fat content and lactation period. The slope A for lactation period was −0.11. The gray area represents the 95% CI of fat content values vs. the lactation period values. B. Negative association between fat content and udder cleanliness. The slope B for udder cleanliness was −0.074. The gray area represents the 95% CI of fat content values vs. udder cleanliness values; Figure S7: Pearson residual distribution of the simplified model for protein content; Figure S8: A. Positive association between protein content and housing. The slope A for housing was 0.21. The gray area represents the 95% CI of protein content values vs. the housing values. B. Positive association between protein content and DIM. The slope B for DIM was 0.001. The gray area represents the 95% CI of protein content values vs. DIM values; Figure S9: A. Negative association between protein content and lactation period. The slope A for lactation period was −0.08. The gray area represents the 95% CI of protein content values vs. the lactation period values. B. Negative association between protein content and udder cleanliness. The slope B for udder cleanliness was −0.06. The gray area represents the 95% CI of protein content values vs. udder cleanliness values; Figure S10: A. Negative association between protein content and teat size. The slope A for teat size was −0.09. The gray area represents the 95% CI of protein content values vs. the teat size values. B. Negative association between protein content and teat hyperkeratosis. The slope B for teat hyperkeratosis was −0.05. The gray area represents the 95% CI of protein content values vs. teat hyperkeratosis values; Figure S11: Pearson residual distribution of the simplified model for lactose content; Figure S12: A. Negative association between lactose content and housing. The slope A for housing was −0.05. The gray area represents the 95% CI of lactose content values vs. the housing values. B. Positive association between lactose content and DMY. The slope B for DMY was 0.01. The gray area represents the 95% CI of lactose content values vs. DMY values; Figure S13: A. Negative association between lactose content and DIM. The slope A for DIM was −0.0002. The gray area represents the 95% CI of lactose content values vs. the DIM values. B. Negative association between lactose content and lactation period. The slope B for lactation period was −0.079. The gray area represents the 95% CI of lactose content values vs. lactation period values; Figure S14: Pearson residual distribution of the simplified model for solid non-fat content; Figure S15: A. Negative association between solid non-fat content and lactation period. The slope A for lactation period was −0.199. The gray area represents the 95% CI of solid non-fat content values vs. the lactation period values. B. Negative association between solid non-fat content and DMY. The slope B for DMY was −0.007. The gray area represents the 95% CI of solid-non-fat values vs. DMY values; Figure S16: A. Negative association between solid non-fat content and udder cleanliness. The slope A for udder cleanliness was −0.04. The gray area represents the 95% CI of solid-non-fat values vs. the udder cleanliness values, and B. Negative association between solid non-fat content and teat hyperkeratosis. The slope B for teat hyperkeratosis was −0.071. The gray area represents the 95% CI of solid non-fat content values vs. teat hyperkeratosis values; Figure S17: Negative association between solid non-fat content and teat size. The slope for teat size was −0.097. The gray area represents the 95% CI of solid-non-fat content values vs. the teat size values; Figure S18: Pearson residual distribution of the simplified model for total solids content; Figure S19: A. Negative association between total solids content and mastitis prevalence. The slope A for mastitis prevalence was −0.23. The gray area represents the 95% CI of total solids content values vs. the mastitis prevalence values. B. Negative association between total solids content and DIM. The slope B for DIM was −0.001. The gray area represents the 95% CI of total solids content values vs. DIM values; Figure S20: A. Negative association between total solids content and udder cleanliness. The slope A for udder cleanliness was −0.09. The gray area represents the 95% CI of total solids content values vs. the udder cleanliness values. B. Negative association between total solids content and lactation period. The slope B for lactation period was −0.25. The gray area represents the 95% CI of total solids content values vs. lactation period values; Figure S21: A. Negative association between total solids content and teat size. The slope A for teat size was −0.19. The gray area represents the 95% CI of total solids content values vs. the teat size values. B. Negative association between total solids content and teat hyperkeratosis. The slope B for teat hyperkeratosis was −0.13. The gray area represents the 95% CI of total solids content values vs. teat hyperkeratosis values.
