# Supplementary figures and images for: A Cross-Sectional Study of Risk Factors Affecting Milk Quality in Dairy Cows
Source: Animals (Basel). 2023 Nov 10;13(22):3470. doi: 10.3390/ani13223470 (PMC10668648; doi:10.3390/ani13223470)

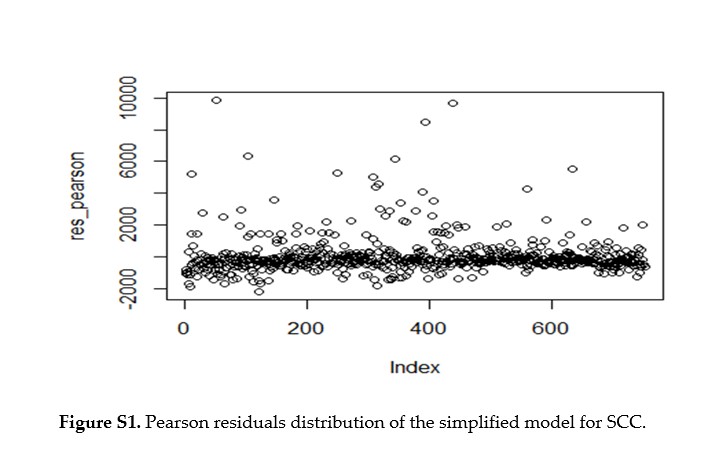

Supplement: Supplementary file 1 [file animals-13-03470-s001.zip › Figure S1.jpg]

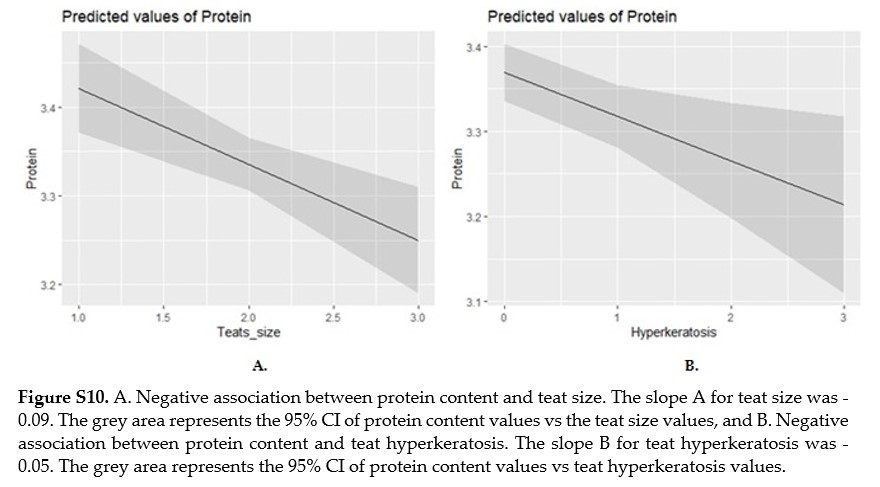

Supplement: Supplementary file 1 [file animals-13-03470-s001.zip › Figure S10.jpg]

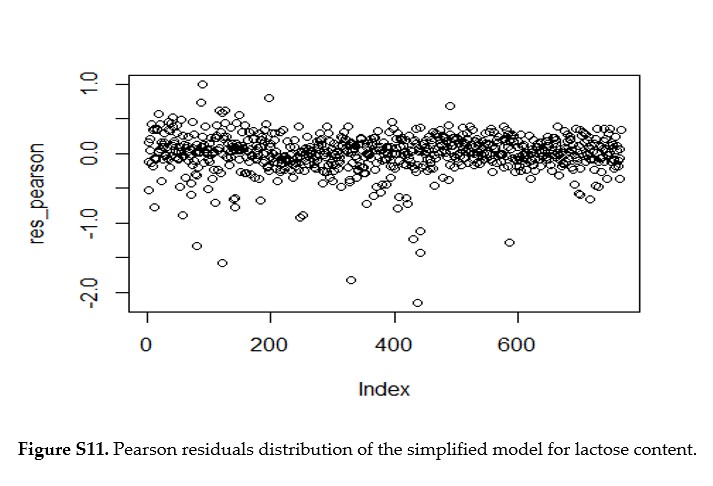

Supplement: Supplementary file 1 [file animals-13-03470-s001.zip › Figure S11.jpg]

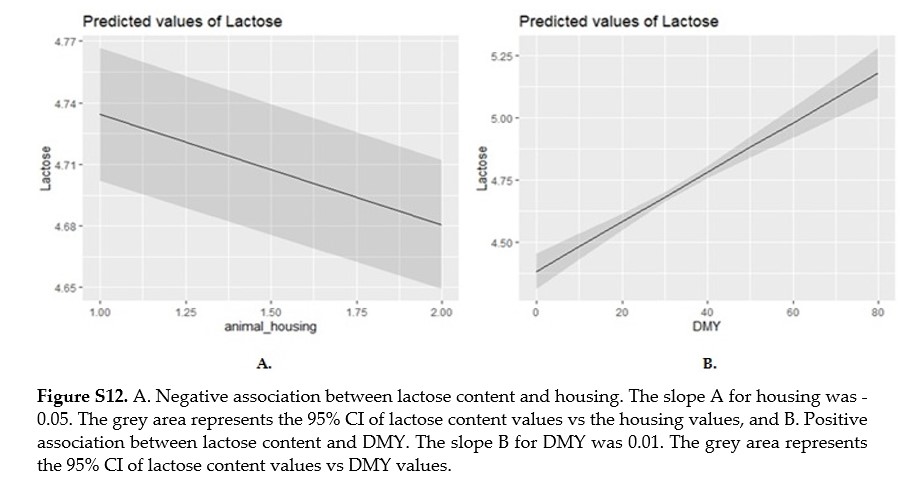

Supplement: Supplementary file 1 [file animals-13-03470-s001.zip › Figure S12.jpg]

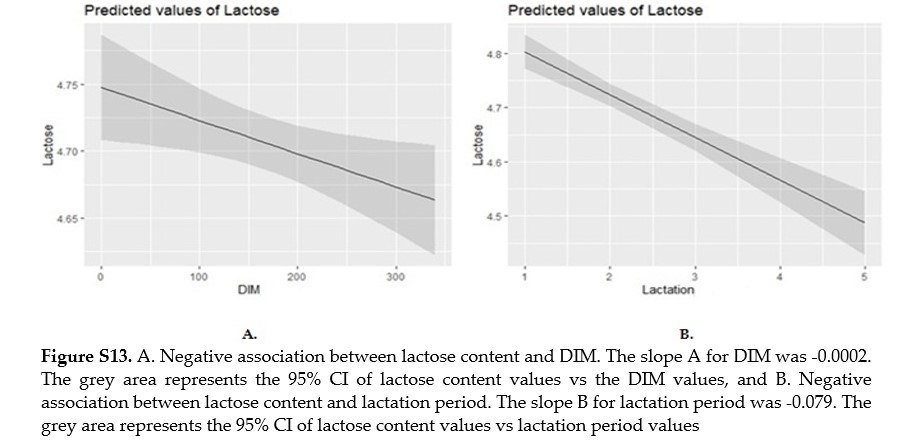

Supplement: Supplementary file 1 [file animals-13-03470-s001.zip › Figure S13.jpg]

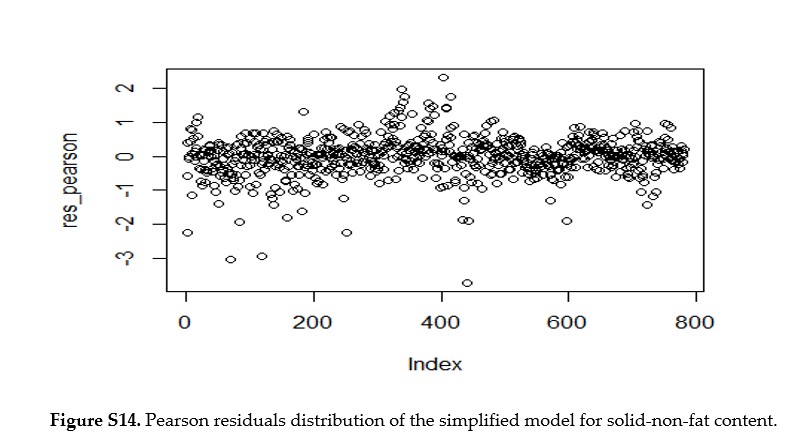

Supplement: Supplementary file 1 [file animals-13-03470-s001.zip › Figure S14.jpg]

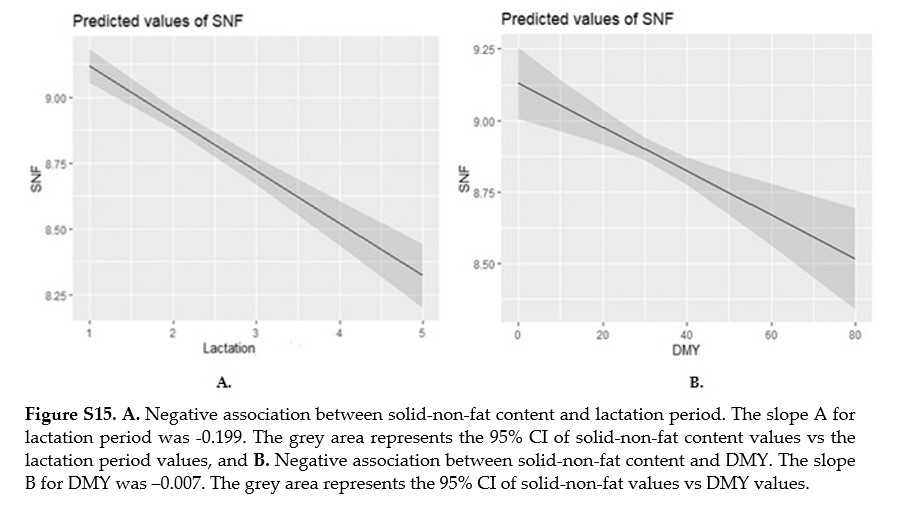

Supplement: Supplementary file 1 [file animals-13-03470-s001.zip › Figure S15.jpg]

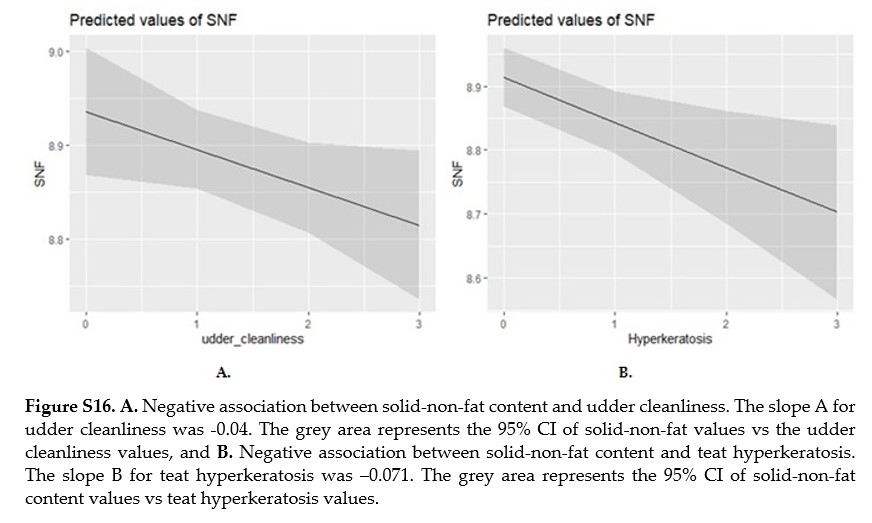

Supplement: Supplementary file 1 [file animals-13-03470-s001.zip › Figure S16.jpg]

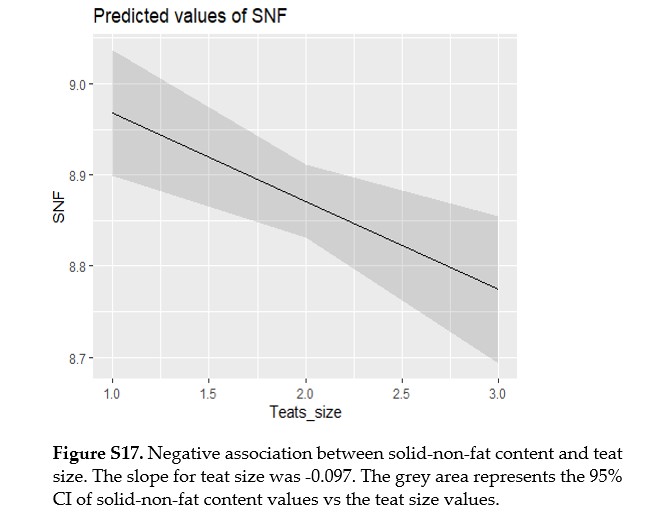

Supplement: Supplementary file 1 [file animals-13-03470-s001.zip › Figure S17.jpg]

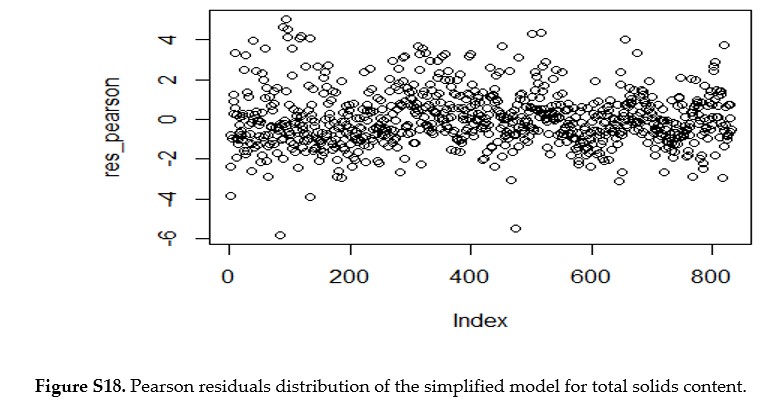

Supplement: Supplementary file 1 [file animals-13-03470-s001.zip › Figure S18.jpg]

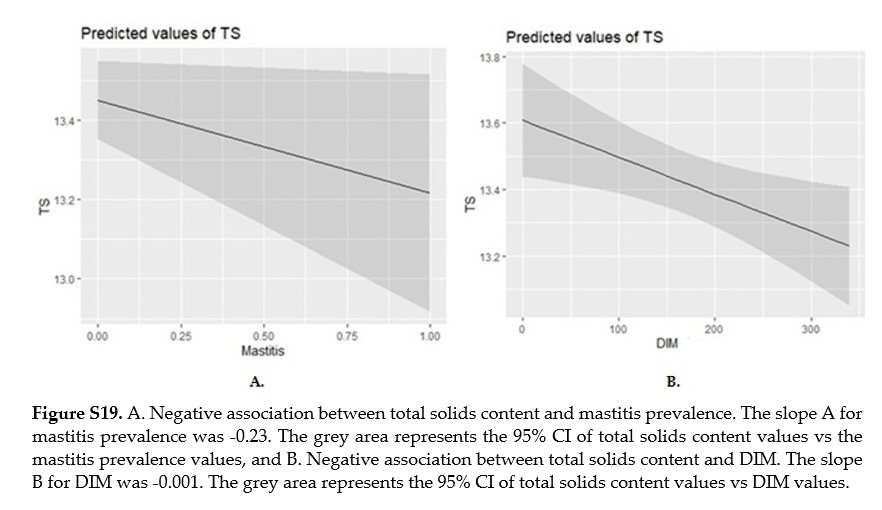

Supplement: Supplementary file 1 [file animals-13-03470-s001.zip › Figure S19.jpg]

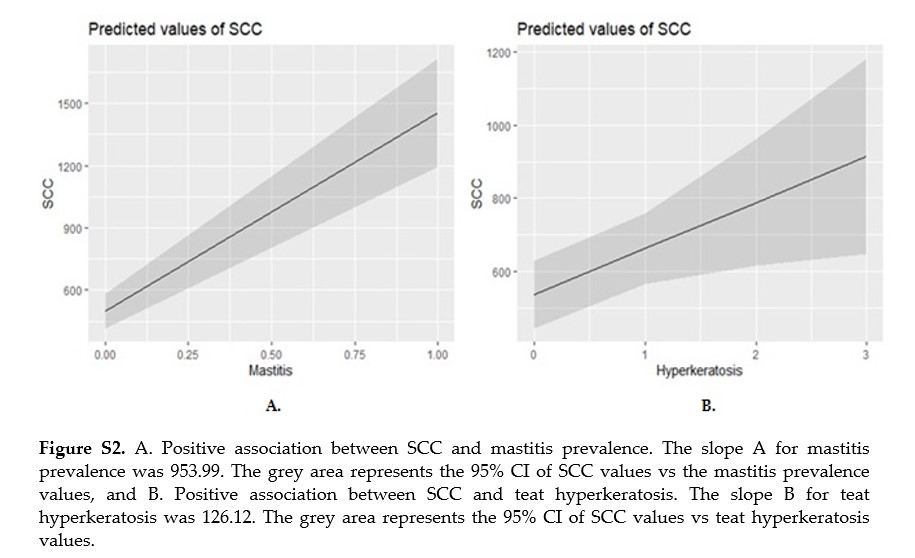

Supplement: Supplementary file 1 [file animals-13-03470-s001.zip › Figure S2.jpg]

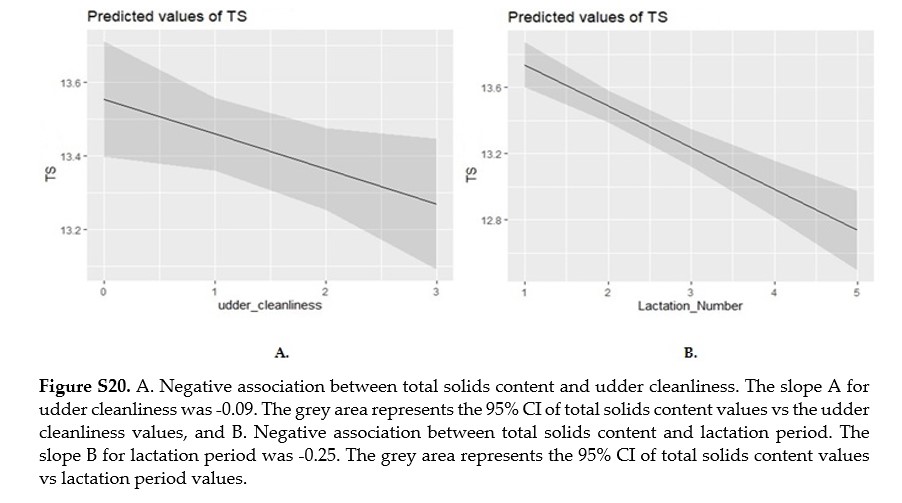

Supplement: Supplementary file 1 [file animals-13-03470-s001.zip › Figure S20.jpg]

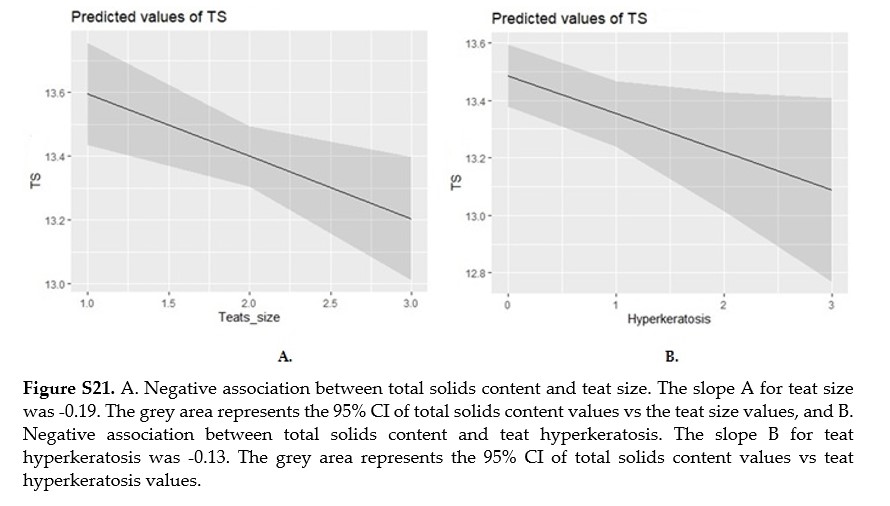

Supplement: Supplementary file 1 [file animals-13-03470-s001.zip › Figure S21.jpg]

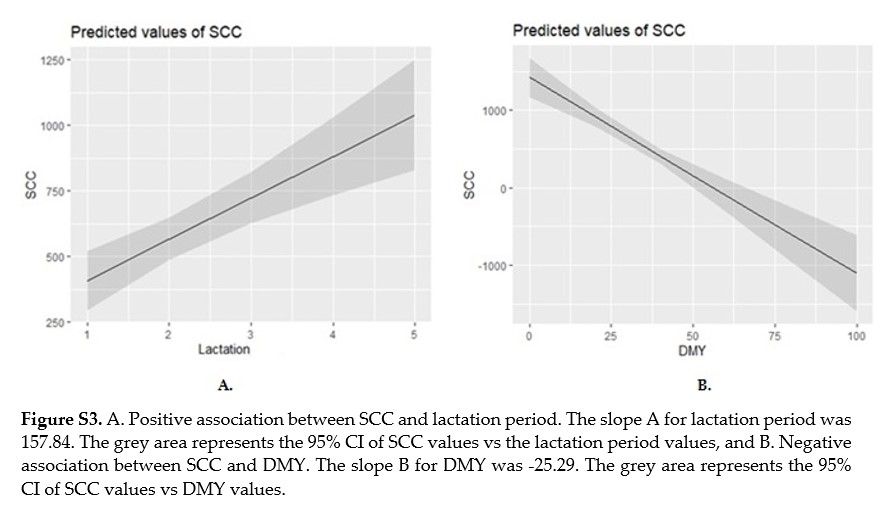

Supplement: Supplementary file 1 [file animals-13-03470-s001.zip › Figure S3.jpg]

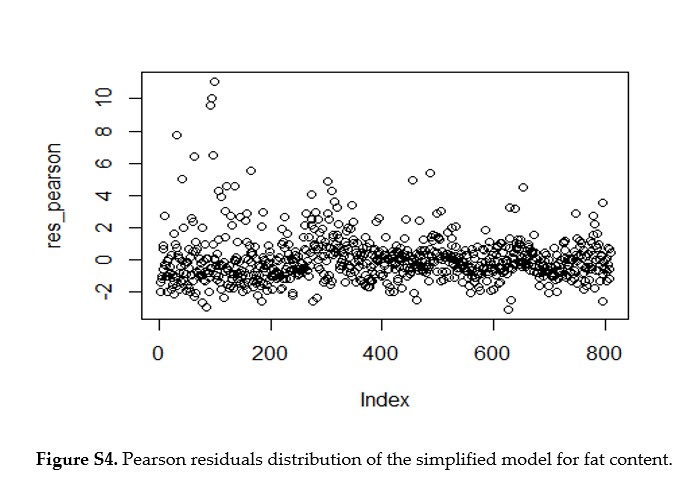

Supplement: Supplementary file 1 [file animals-13-03470-s001.zip › Figure S4.jpg]

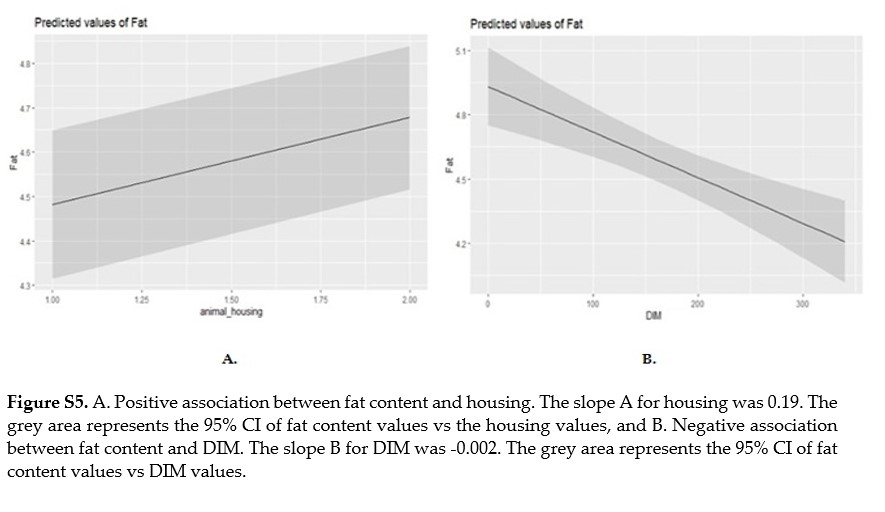

Supplement: Supplementary file 1 [file animals-13-03470-s001.zip › Figure S5.jpg]

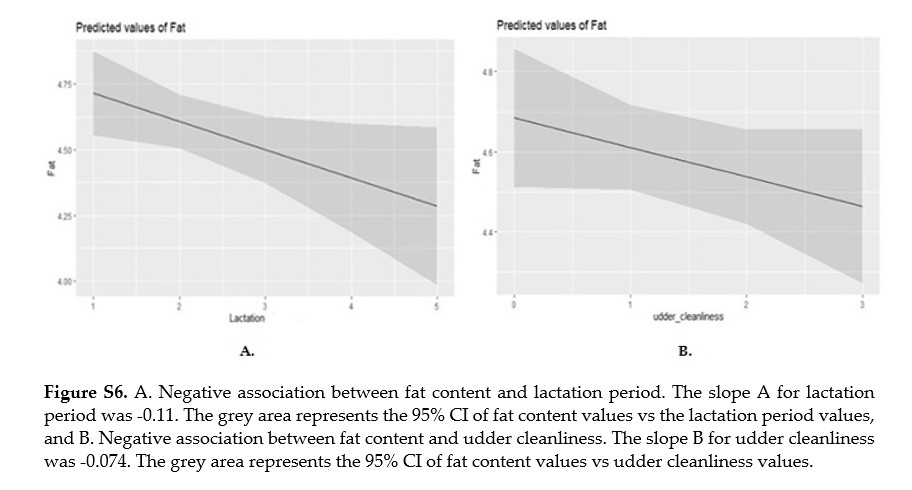

Supplement: Supplementary file 1 [file animals-13-03470-s001.zip › Figure S6.jpg]

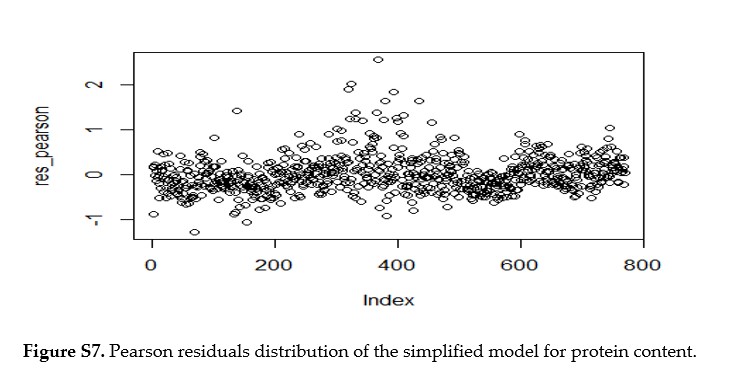

Supplement: Supplementary file 1 [file animals-13-03470-s001.zip › Figure S7.jpg]

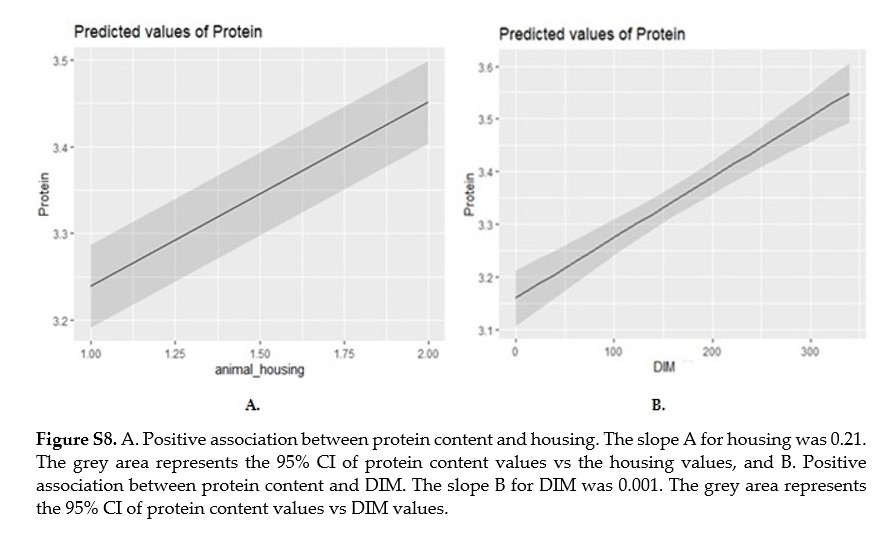

Supplement: Supplementary file 1 [file animals-13-03470-s001.zip › Figure S8.jpg]

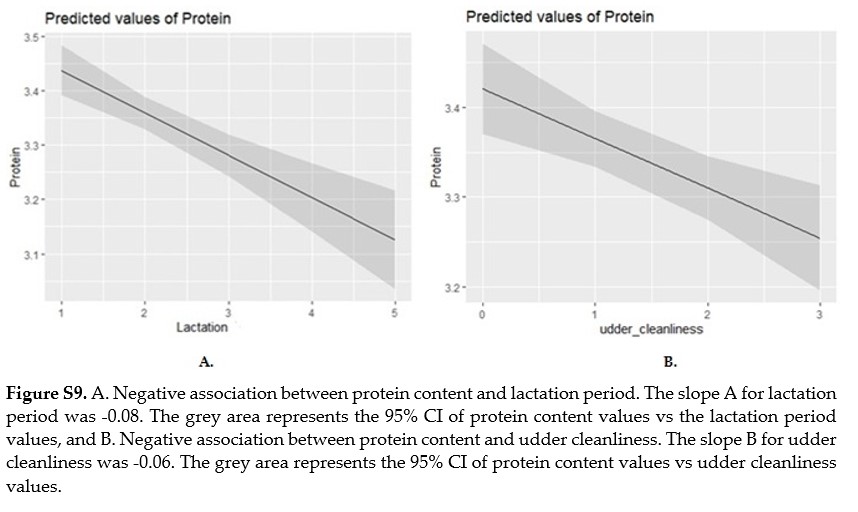

Supplement: Supplementary file 1 [file animals-13-03470-s001.zip › Figure S9.jpg]

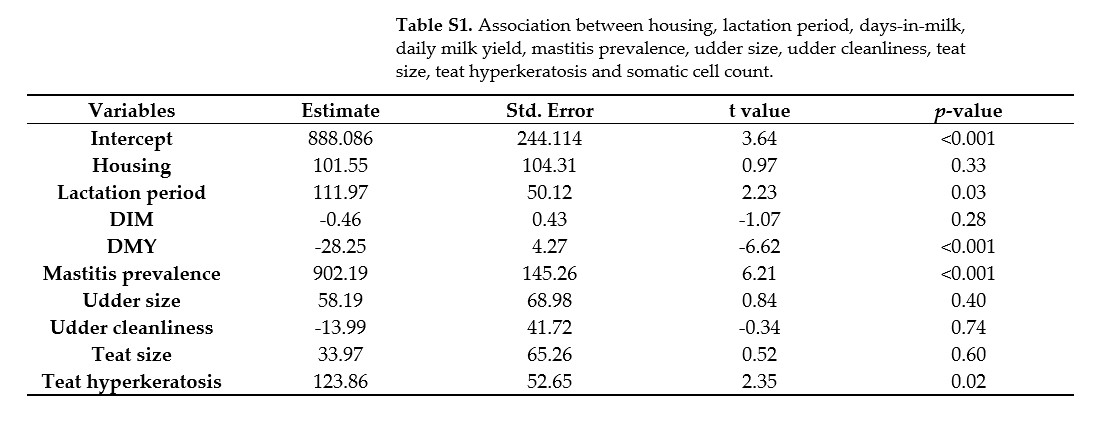

Supplement: Supplementary file 1 [file animals-13-03470-s001.zip › Table S1.jpg]

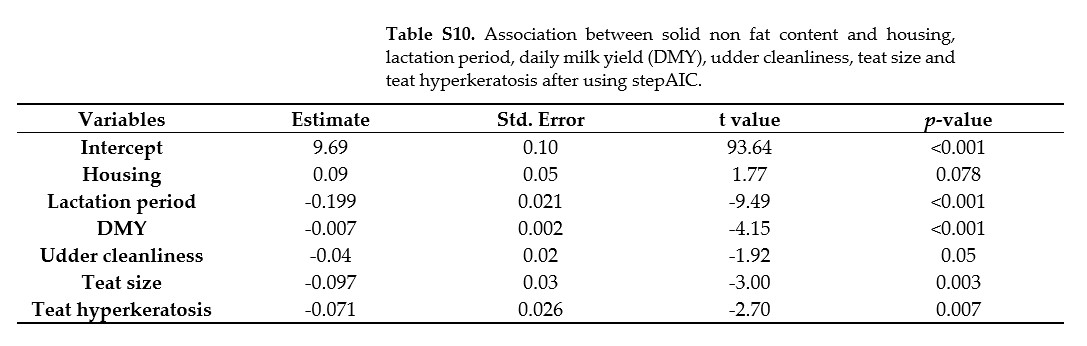

Supplement: Supplementary file 1 [file animals-13-03470-s001.zip › Table S10.jpg]

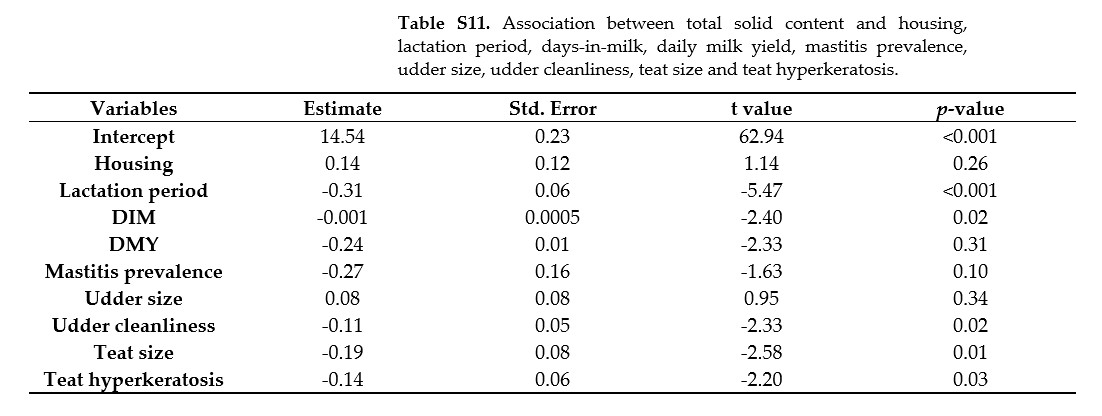

Supplement: Supplementary file 1 [file animals-13-03470-s001.zip › Table S11.jpg]

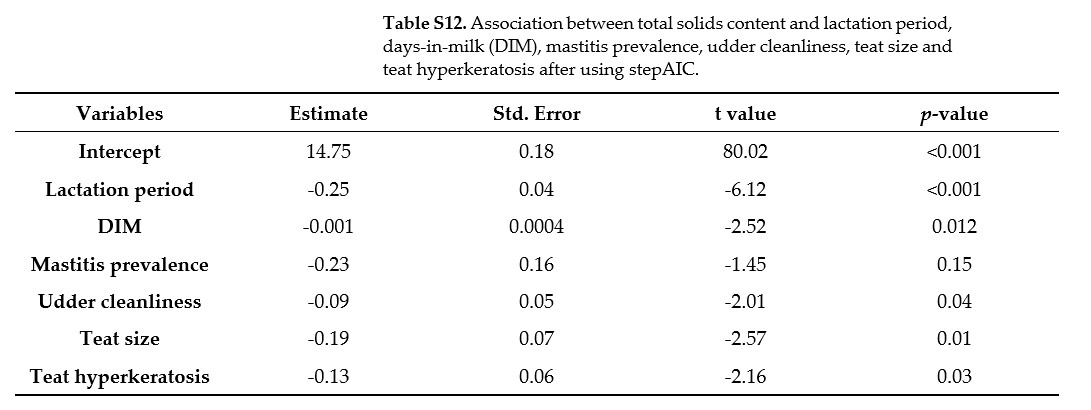

Supplement: Supplementary file 1 [file animals-13-03470-s001.zip › Table S12.jpg]

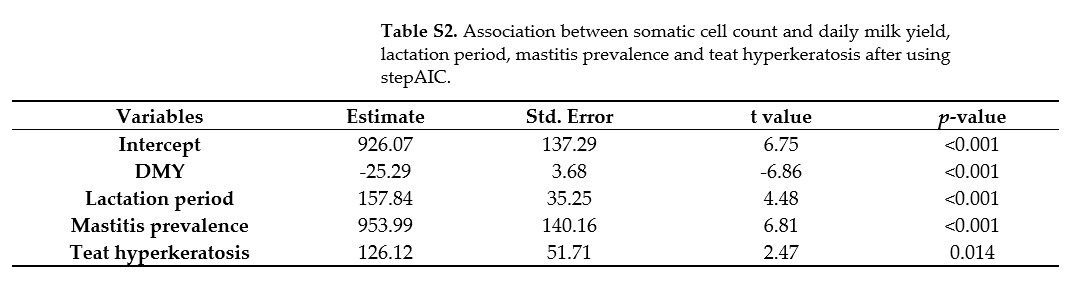

Supplement: Supplementary file 1 [file animals-13-03470-s001.zip › Table S2.jpg]

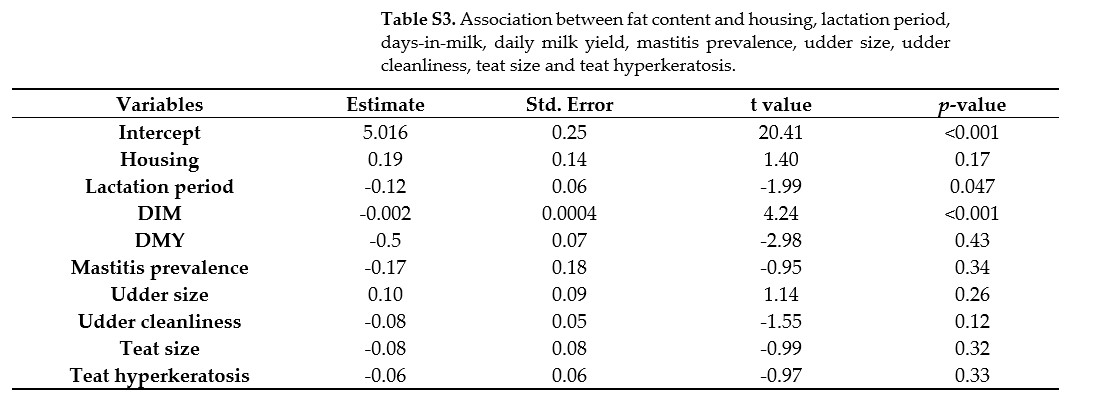

Supplement: Supplementary file 1 [file animals-13-03470-s001.zip › Table S3.jpg]

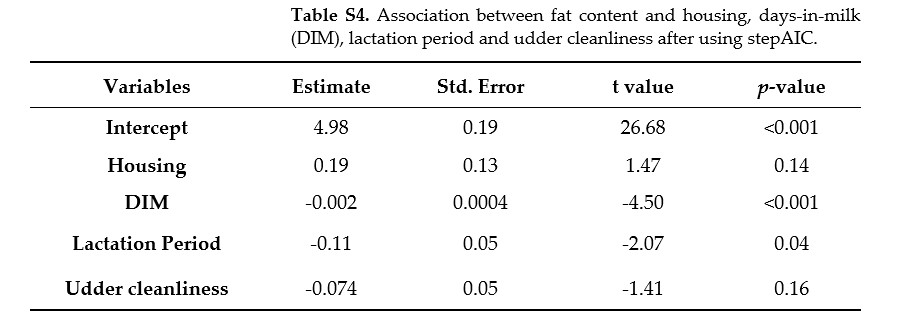

Supplement: Supplementary file 1 [file animals-13-03470-s001.zip › Table S4.jpg]

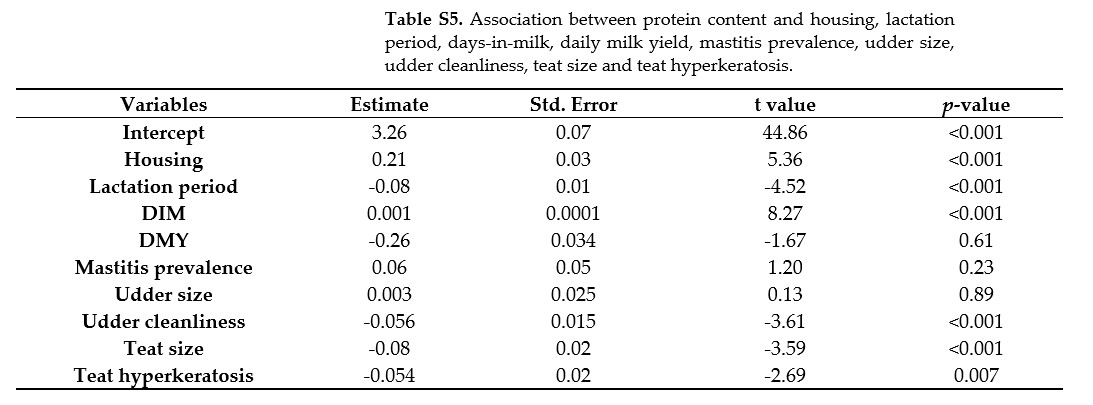

Supplement: Supplementary file 1 [file animals-13-03470-s001.zip › Table S5.jpg]

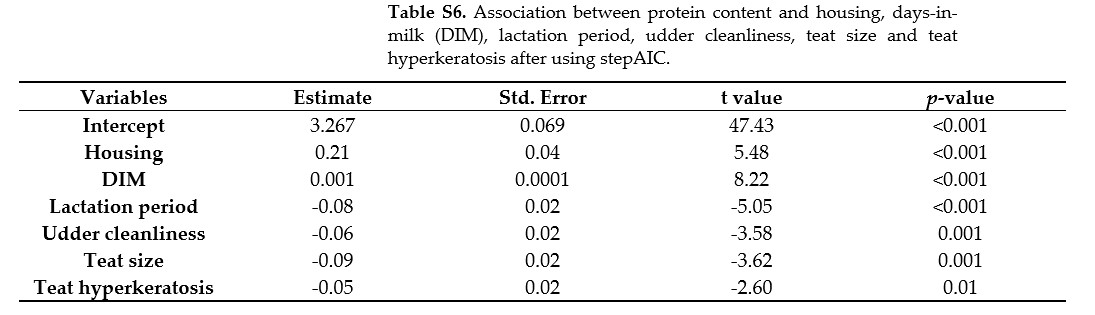

Supplement: Supplementary file 1 [file animals-13-03470-s001.zip › Table S6.jpg]

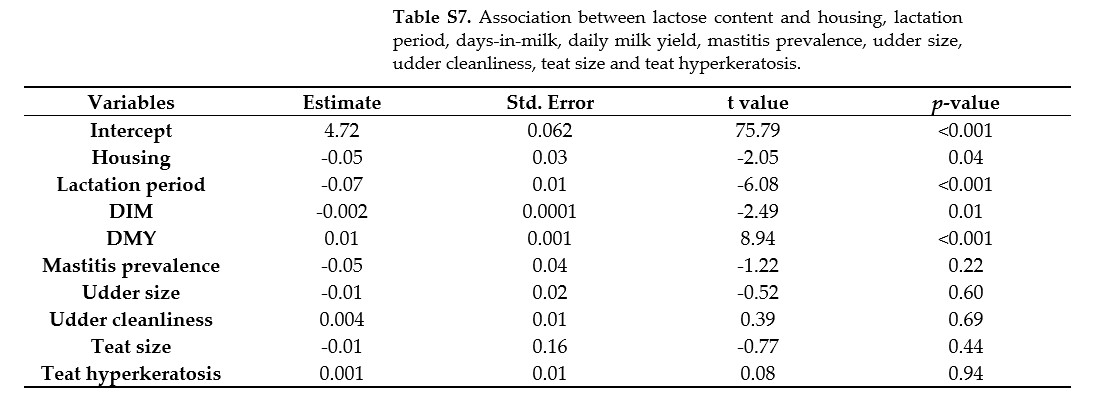

Supplement: Supplementary file 1 [file animals-13-03470-s001.zip › Table S7.jpg]

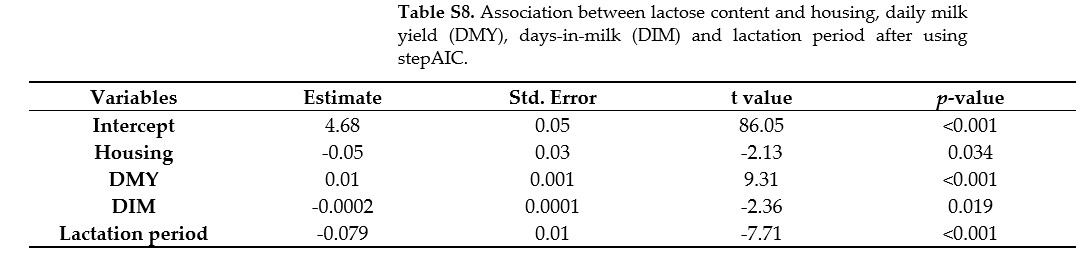

Supplement: Supplementary file 1 [file animals-13-03470-s001.zip › Table S8.jpg]

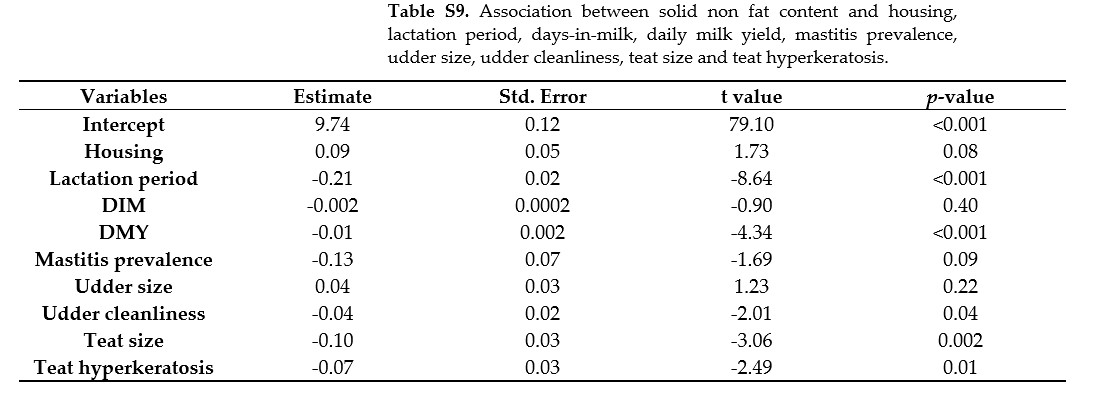

Supplement: Supplementary file 1 [file animals-13-03470-s001.zip › Table S9.jpg]
